# What is a good result after clubfoot treatment? A Delphi-based consensus on success by regional clubfoot trainers from across Africa

**DOI:** 10.1371/journal.pone.0190056

**Published:** 2017-12-21

**Authors:** Tracey Smythe, Andrew Wainwright, Allen Foster, Christopher Lavy

**Affiliations:** 1 International Centre for Evidence in Disability, London School of Hygiene and Tropical Medicine, London, United Kingdom; 2 Nuffield Department of Orthopaedics Rheumatology and Musculoskeletal Science, University of Oxford, Headington, United Kingdom; Kanazawa University, JAPAN

## Abstract

**Background:**

Congenital talipes equino-varus (CTEV), also known as clubfoot, is one of the most common congenital musculoskeletal malformations. Despite this, considerable variation exists in the measurement of deformity correction and outcome evaluation. This study aims to determine the criteria for successful clubfoot correction using the Ponseti technique in low resource settings through Africa.

**Methods:**

Using the Delphi method, 18 experienced clubfoot practitioners and trainers from ten countries in Africa ranked the importance of 22 criteria to define an ‘acceptable or good clubfoot correction’ at the end of bracing with the Ponseti technique. A 10cm visual analogue scale was used. They repeated the rating with the results of the mean scores and standard deviation of the first test provided. The consistency among trainers was determined with the intra-class correlation coefficient (ICC). From the original 22 criteria, ten criteria with a mean score >7 and SD <2 were identified and were rated through a second Delphi round by 17 different clubfoot treatment trainers from 11 countries in Africa. The final definition consisted of all statements that achieved strong agreement, a mean score of >9 and SD<1.5.

**Results:**

The consensus definition of a successfully treated clubfoot includes: (1) a plantigrade foot, (2) the ability to wear a normal shoe, (3) no pain, and (4) the parent is satisfied. Participants demonstrated good consistency in rating these final criteria (ICC 0.88; 0.74,0.97).

**Conclusions:**

The consistency of Ponseti technique trainers from Africa in rating criteria for a successful outcome of clubfoot management was good. The consensus definition includes basic physical assessment, footwear use, pain and parent satisfaction.

## Introduction

Congenital talipes equino-varus (CTEV), or clubfoot, is one of the most prevalent congenital musculoskeletal malformations that affects mobility [1]. The most common method of treatment worldwide is now the Ponseti technique [2]. This primarily non-operative technique is beneficial in low- and middle-income countries (LMICs) where there are limited resources and different cadres of health workers can be trained to treat clubfoot [3]. Despite this, considerable variation exists in the assessment of deformity correction and outcome. Goals of clubfoot treatment include improvement in foot function, the creation of a pain free, shoe-able foot, parent satisfaction [4,5] and avoidance of corrective surgery [6]. As the treatment can be delivered by trained health workers [7], the non-specialist health workers require valid, repeatable and easy to measure outcome measures to determine their results of clubfoot treatment in low-resource settings. There is no consensus regarding the definition of success of clubfoot management and diverse criteria [8–10] have been proposed. The concept of success after the bracing phase requires further investigation in environments that share a context of public health systems with overcrowded clinics and limited access to equipment, such as goniometers.

The Delphi method is a structured consensus technique that may be used to reach agreement about outcomes [11]. It is a sequential process through which the anonymous opinions of participants are sought [12] and this allows equal weight to be given to all participants [13,14]. After the completion of each round of questionnaires, the collated group responses are fed back to participants. Establishing consensus does not ensure validity, however agreement provides a basis for establishing criteria that are likely to have clinical sensibility [15].

This study aims to determine criteria for successful clubfoot correction at the conclusion of the bracing phase in a low resource setting, by establishing consensus amongst expert Ponseti trainers in the Africa region.

## Materials and methods

The study was performed and reported following the recommended guidelines [11] for selection of healthcare quality indicators. Eighteen trainers from ten national clubfoot programmes in Africa attended a workshop in January 2016. The trainers were regional experts in clubfoot management, and they deliver training in the Ponseti method in their respective countries. The participants were chosen based on willingness to participate and knowledge of the topic [16] and included orthopaedic surgeons, physiotherapists and orthopaedic technicians. The mean length of time that the trainers had used the Ponseti method for was 7.7 years (95%CI 6.0–9.3) and the average number of trainings delivered was 4.7 (95%CI 2.2–7.1)

The Delphi method employed was a two-round self-administered questionnaire. To identify outcomes that are important, the questionnaire was developed through a regional workshop of Ponseti experts [17]. Potential criteria for assessment of good clubfoot correction were discussed. A systematic literature review of outcomes reported for clubfoot treatment through Africa found different definitions of success at various points in clubfoot treatment [18–23], all of which were discussed by the experts. Twenty-two potentially relevant criteria for good clubfoot correction were identified in the workshop by the Ponseti technique trainers. The questionnaire was pilot tested for suitability.

The 18 regional trainers were invited to participate, and all completed a questionnaire that included the criteria of a successful outcome, previously generated in the workshop. The questionnaire asked the respondents to rate each of the items for their relative importance using a 10cm visual analogue scale (VAS) with the anchors ‘completely unimportant’ and ‘extremely important’ at each extreme. Respondents were asked to consider all the listed criteria as independent; the paper questionnaires were completed by hand.

The VAS means and standard deviations (SD) were calculated based on the responses of all the trainers. After two days, a second questionnaire was delivered to the same 18 trainers with the results of the previous questionnaire (VAS mean and SD). No criteria were excluded and there was no discussion among the participants.

The consistency among the 18 trainers was determined with the intra-class correlation coefficient (ICC). The conventional interpretation of the ICC is as follows: ≤0.40, poor consistency or large variation in opinion; 0.41 to 0.74, acceptable consistency; and ≥0.75 good consistency [24]. All data were managed and analysed using Stata 14.2, StataCorp 4905, Lakeway Drive College Station, Texas 77845, USA.

In July 2016, 6 months after the first workshop, a second, two-round Delphi method was used to reach a consensus definition of good or acceptable clubfoot correction after bracing. Ten “successful outcome” criteria generated in January 2016 were included which met two predefined criteria: (1) a mean VAS higher than 7 on the ten-point scale, and SD <2; and (2) applicable to outcome at the conclusion of the bracing phase. As there is variability in the measurement of distribution of scores in studies that use the Delphi method [25], the thresholds for the VAS mean and SD were decided *a-priori*. The aim was to generate a list of items that participants considered important to assess for acceptable clubfoot correction after bracing from the original list of questions that related to outcome in general.

Seventeen different regional clubfoot trainers (from eleven countries) who attended a workshop in July 2016 were invited to participate. The mean length of time that the trainers had used the Ponseti method for was 7.9 years (95%CI 6.9–9.9) and the average number of trainings delivered was 8.3 (95%CI 5.1–11.5). The Delphi process was similar to that undertaken in January using a 10cm visual analogue scale with the anchors ‘completely unimportant’ and ‘extremely important.’ The paper questionnaires were completed by hand. The trainers repeated the rating with the results of the mean scores and standard deviation of the first test visible two days later. The questionnaires used in the second rounds of the Delphi process are included in Supplementary Information files (S1 and S2 Files).

Criteria with a mean VAS rated > 9 and with a SD < 1.5 were considered to have high agreement. Where two criteria described the same indicator with the difference being only the language used, (e.g. foot is flat on the floor and plantigrade foot) the criterion with the highest VAS mean and lowest SD was selected.

**Fig 1:**
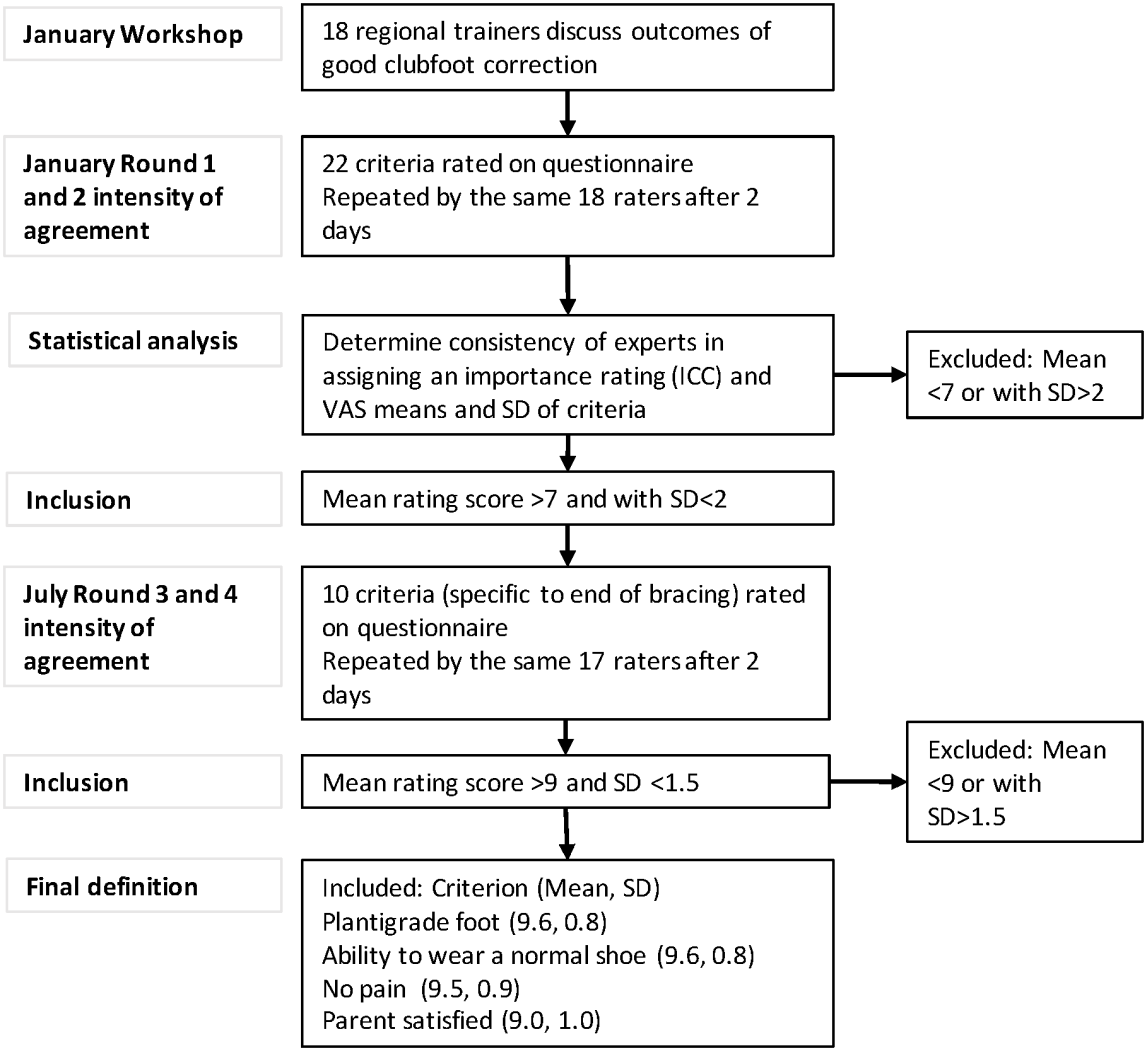
Flow chart of criteria selection. Definitions of abbreviations: SD = standard deviation, ICC = intra-class correlation, VAS = visual analogue scale).

The study methodology and course of action for the management of responses is outlined in Fig 1.

### Ethics statement

Ethical approval for this study was granted by the London School of Hygiene & Tropical Medicine Ethics Committee (approval number 10412). Written consent was obtained at the beginning of the workshop and data were analysed anonymously.

## Results

The response rate of trainers to the questionnaires was 100% on the first round and 94% on the second round in both January and July 2016. The consistency of Ponseti trainers in Africa in rating criteria for successful outcome of clubfoot management was good. The first Delphi ICC had external consistency of 0.83 (0.71–0.92) and the second Delphi ICC had external consistency of 0.88 (0.74–0.97). From the initial 22 criteria, 10 met the inclusion criteria for the second two rounds of Delphi. Details for the ranking of each criterion by trainers in Africa are shown in Tables 1 and 2.

**Table 1:** Final rating of criteria in the first Delphi round (ordered by visual analogue scale mean and standard deviation).

First Delphi round, January 2016		
Criterion	Mean	SD
The foot fits comfortably into a Foot Abduction Brace	8.90	0.71
The foot is plantigrade	8.78	1.26
The foot has 15 degrees of dorsiflexion or more	8.57	1.95
The heel is in a neutral position (no longer in varus)	8.23	1.53
The child can wear a normal shoe	8.16	1.76
The child reports no pain	8.13	1.83
The child demonstrates heel strike when walking	8.11	1.64
The forefoot adductus is corrected	8.09	1.15
The carer is satisfied	7.96	1.43
The foot does not supinate in swing phase when walking	7.92	1.33
The foot does not have less than 60 degrees of abduction	7.74	1.86
The Pirani score is 0.5 or less	7.67	3.04
The child keeps up with peers when walking and running	7.58	2.24
The foot has 10 degrees of dorsiflexion or more	7.52	1.81
The Pirani score is 0.5 or less	6.90	2.73
The foot is corrected within 6 casts	6.76	2.54
The Pirani score is 1 or less	6.66	2.72
The wear on the shoes are symmetrical (in unilateral clubfoot)	6.10	2.67
The child had a tenotomy	5.80	3.02
The foot has more than 30 degrees of abduction	5.44	1.74
The Pirani score is 1.5 or less	5.36	2.34
The Pirani score is 2 or less	4.94	2.74

**Table 2:** Final rating of criteria in the second Delphi round (ordered by visual analogue scale mean and standard deviation).

Second Delphi round, July 2016		
Criterion	Mean	SD
The foot is plantigrade	9.56	0.79
The child can wear a normal shoe	9.56	0.79
The child reports no pain	9.47	0.88
The carer is satisfied	9.01	1.13
The foot has 15 degrees of dorsiflexion or more	8.99	1.01
The heel is in a neutral position (no longer in varus)	8.79	1.10
The forefoot adductus is corrected	8.65	1.22
The child demonstrates heel strike when walking	8.51	1.01
The foot does not supinate in swing phase when walking	8.44	1.18
The foot does not have less than 60 degrees of abduction	7.44	2.04

**Fig 2:**
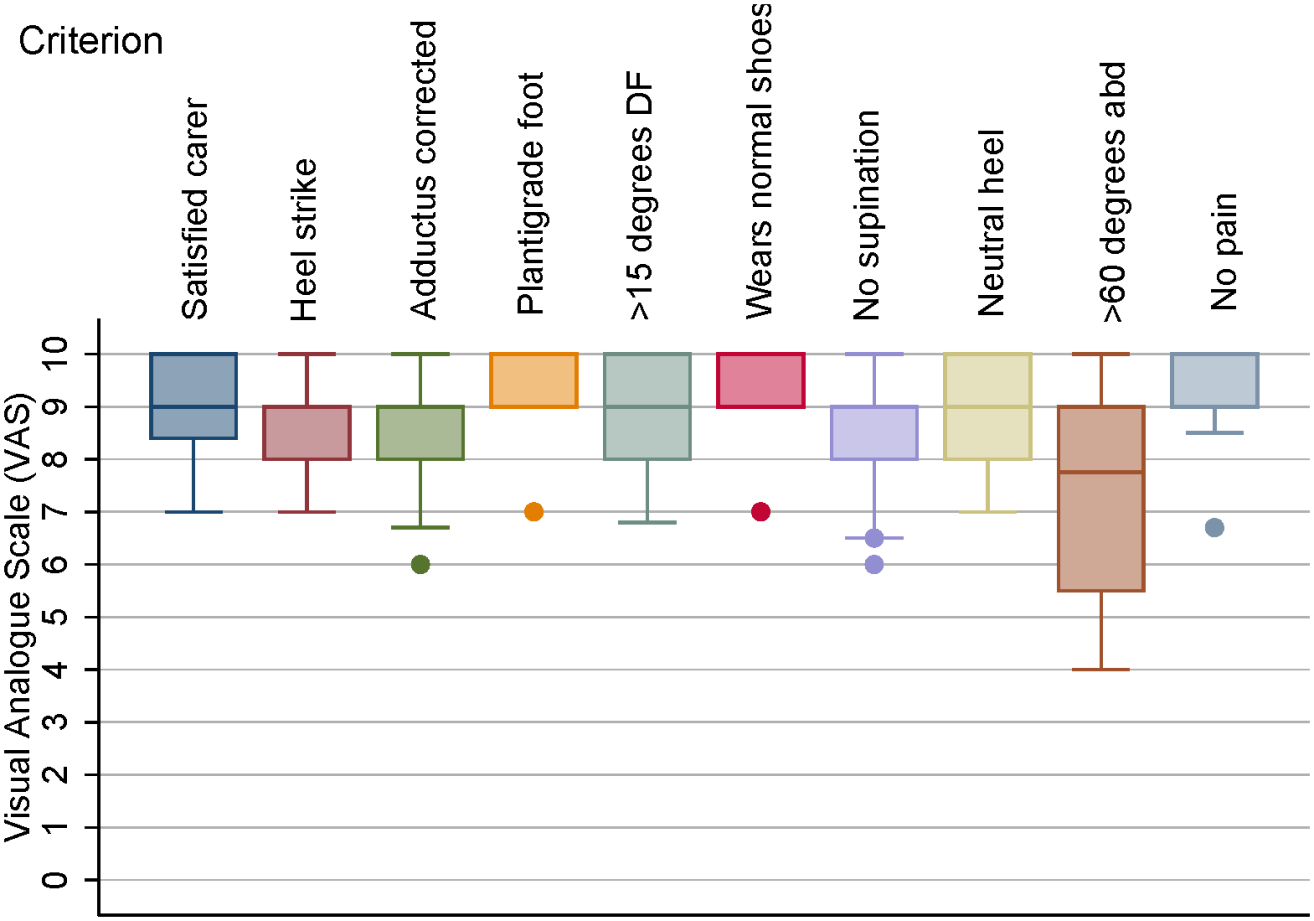
Ratings for successful clubfoot correction after bracing (10 criteria in the order asked on the questionnaire). Box and whisker plot of the final ten criteria. The middle 50% of the VAS ratings are shown as the box. The horizontal line in the box represents the median value. The upper and lower quartiles are indicated by the whiskers and outliers are indicated by a circle. (Definitions of abbreviations: DF = dorsiflexion, abd = abduction).

The distribution of the data for the final ten criteria is displayed in Fig 2.

Outcomes that had >9 VAS mean with <1.5 SD were criterion (4) plantigrade foot, (6) ability to wear a normal shoe, (10) no pain and (1) carer is satisfied.

## Discussion

Non-specialist health workers require valid, repeatable and easy to measure outcome measures to determine their results of clubfoot treatment in clinics through Africa. This study determined the opinions of experts from eleven countries in Africa about the criteria for success following clubfoot treatment after the manipulation and bracing phases with the Ponseti technique. The aim of the Delphi method was to define criteria that could be used by any clubfoot practitioner working in busy clinics with limited resources through Africa. Regional trainers were therefore deemed the most appropriate experts to interview in this context.

This study found that the highest rated outcomes were a plantigrade foot, ability to wear a normal shoe, parent satisfaction and absence of pain. These criteria are included in other published assessment tools. Laaveg and Ponseti described a detailed functional rating system [8] that requires the use of a goniometer to evaluate outcomes of treatment and incorporates patient satisfaction, pain, gait, heel position and range of motion. The Roye tool [4] consists of ten questions designed to measure treatment outcomes through overall satisfaction, appearance, pain and physical limitations in a high income setting [26]. The Bangla tool [9] was developed to evaluate results of clubfoot management in Bangladesh, where clinics required a tool that was quick, relevant and reliable for use in children of walking age. The Clubfoot Assessment Protocol (CAP) includes a detailed assessment of movement quality and requires accurate passive mobility testing with a goniometer and awareness of muscle testing [10], but it does not include parent reported outcomes. These four tools have been developed in local contexts by individual institutions.

This study used a Delphi process with many experts, in the context of Africa, to develop and then rank criteria that are viewed to be important in the assessment of a successful outcome for clubfoot management in low resource settings. The finding that these four criteria are included in the criteria of the other published assessment tools contributes to evidence of their validity.

There are limitations of this study. Previous research has shown that panel composition influences ratings [27]. The panel in this study was selected for their expertise, but may not be representative of all Ponseti treatment practitioners. There may be some criteria that were not considered which may also be important.

The expert trainers showed good consistency in rating satisfactory outcomes for clubfoot management (ICC 0.88; 0.74,0.97) and a strength of this study includes the high response rate of the survey (94%).

The consensus definition includes four criteria—a simple physical assessment, footwear use, patient pain and parent reported outcome measures. It is likely that these four criteria will provide a good overall assessment of successful treatment of a child with clubfoot in Africa, and may be useful in other geographic contexts after further investigation. The use of these four criteria should allow the development a simple assessment tool that can be used by non-specialist health workers. The other aspects of utility and reliability of this tool will then need to be studied in future research.

## Conclusion

Appropriate measures are required to determine the successful outcome of clubfoot treatment and to compare different treatment techniques in low resource settings. Using a Delphi process with experts from across Africa, we were able to find consensus for the four most important criteria of a successful clubfoot treatment using the Ponseti method.

## Supporting information

S1 File Questionnaire for Delphi 1 round 2. (DOCX)

S2 File Questionnaire for Delphi 2 round 2. (DOCX)
