# Supplementary material for: What is a good result after clubfoot treatment? A Delphi-based consensus on success by regional clubfoot trainers from across Africa
Source: PLoS One. 2017 Dec 21;12(12):e0190056. doi: 10.1371/journal.pone.0190056 (PMC5739468; doi:10.1371/journal.pone.0190056)
Supplement: S1 File — (DOCX) [file pone.0190056.s001.docx]

How important are the following in determining a ‘**good’** clubfoot correction?

1. The carer/family is happy with the results (average 8.0)

Ι Ι Ι Ι Ι Ι Ι Ι Ι Ι Ι

0 1 2 3 4 5 6 7 8 9 10

2. The child demonstrates heel strike when walking (average 8.1)

Ι Ι Ι Ι Ι Ι Ι Ι Ι Ι Ι

0 1 2 3 4 5 6 7 8 9 10

3. The forefoot adductus is corrected (average 8.2)

Ι Ι Ι Ι Ι Ι Ι Ι Ι Ι Ι

0 1 2 3 4 5 6 7 8 9 10

4. The wear on the shoes are symmetrical (in unilateral clubfoot) (average 6.1)

Ι Ι Ι Ι Ι Ι Ι Ι Ι Ι Ι

0 1 2 3 4 5 6 7 8 9 10

5. The child had a tenotomy (average 4.1)

Ι Ι Ι Ι Ι Ι Ι Ι Ι Ι Ι

0 1 2 3 4 5 6 7 8 9 10

6. The foot is plantigrade (average 8.7)

Ι Ι Ι Ι Ι Ι Ι Ι Ι Ι Ι

0 1 2 3 4 5 6 7 8 9 10

7. The Pirani score is 2 or less (average 4.1)

Ι Ι Ι Ι Ι Ι Ι Ι Ι Ι Ι

0 1 2 3 4 5 6 7 8 9 10

8. The foot has 15 degrees of dorsiflexion or more (average 8)

Ι Ι Ι Ι Ι Ι Ι Ι Ι Ι Ι

0 1 2 3 4 5 6 7 8 9 10

9. The Pirani score is 1 or less (average 6.3)

Ι Ι Ι Ι Ι Ι Ι Ι Ι Ι Ι

0 1 2 3 4 5 6 7 8 9 10

10. The child can wear a normal shoe (average 8.1)

Ι Ι Ι Ι Ι Ι Ι Ι Ι Ι Ι

0 1 2 3 4 5 6 7 8 9 10

11. The Pirani score is 1.5 or less (average 5.5)

Ι Ι Ι Ι Ι Ι Ι Ι Ι Ι Ι

0 1 2 3 4 5 6 7 8 9 10

12. The foot does not supinate in swing phase when walking (average 7.4)

Ι Ι Ι Ι Ι Ι Ι Ι Ι Ι Ι

0 1 2 3 4 5 6 7 8 9 10

13. The Pirani score is 0.5 or less (average 6.5)

Ι Ι Ι Ι Ι Ι Ι Ι Ι Ι Ι

0 1 2 3 4 5 6 7 8 9 10

14. The Pirani score is 0 (average 6.7)

Ι Ι Ι Ι Ι Ι Ι Ι Ι Ι Ι

0 1 2 3 4 5 6 7 8 9 10

15. The heel is in a neutral position (no longer in varus) (average 7.7)

Ι Ι Ι Ι Ι Ι Ι Ι Ι Ι Ι

0 1 2 3 4 5 6 7 8 9 10

16. The foot is corrected within 6 casts (average 5.6)

Ι Ι Ι Ι Ι Ι Ι Ι Ι Ι Ι

0 1 2 3 4 5 6 7 8 9 10

17. The foot fits comfortably into a foot abduction brace (average 8.8)

Ι Ι Ι Ι Ι Ι Ι Ι Ι Ι Ι

0 1 2 3 4 5 6 7 8 9 10

18. The foot does not have less than 60degrees of abduction (average 7.1)

Ι Ι Ι Ι Ι Ι Ι Ι Ι Ι Ι

0 1 2 3 4 5 6 7 8 9 10

19. The foot has more than 30 degrees of abduction (average 5.7)

Ι Ι Ι Ι Ι Ι Ι Ι Ι Ι Ι

0 1 2 3 4 5 6 7 8 9 10

20. The foot has 15 degrees of dorsiflexion or more (average 7.8)

Ι Ι Ι Ι Ι Ι Ι Ι Ι Ι Ι

0 1 2 3 4 5 6 7 8 9 10

21. The child keeps up with peers when walking and running (average 7.3)

Ι Ι Ι Ι Ι Ι Ι Ι Ι Ι Ι

0 1 2 3 4 5 6 7 8 9 10

22. The child reports no pain (average 7.7)

Ι Ι Ι Ι Ι Ι Ι Ι Ι Ι Ι

0 1 2 3 4 5 6 7 8 9 10
