# Supplementary material for: What is a good result after clubfoot treatment? A Delphi-based consensus on success by regional clubfoot trainers from across Africa
Source: PLoS One. 2017 Dec 21;12(12):e0190056. doi: 10.1371/journal.pone.0190056 (PMC5739468; doi:10.1371/journal.pone.0190056)
Supplement: S2 File — (DOCX) [file pone.0190056.s002.docx]

How important are the following to determine an acceptable or ‘**good’** clubfoot correction at completion of bracing?

1. The carer/family is happy with the results (average 8.9)

Ι Ι Ι Ι Ι Ι Ι Ι Ι Ι Ι

0 1 2 3 4 5 6 7 8 9 10

2. The child demonstrates heel strike when walking (average 8.1)

Ι Ι Ι Ι Ι Ι Ι Ι Ι Ι Ι

0 1 2 3 4 5 6 7 8 9 10

3. The forefoot adductus is corrected (average 8.3)

Ι Ι Ι Ι Ι Ι Ι Ι Ι Ι Ι

0 1 2 3 4 5 6 7 8 9 10

4. The foot is plantigrade (average 9.3)

Ι Ι Ι Ι Ι Ι Ι Ι Ι Ι Ι

0 1 2 3 4 5 6 7 8 9 10

5. The foot has 15 degrees of dorsiflexion or more (average 8.8)

Ι Ι Ι Ι Ι Ι Ι Ι Ι Ι Ι

0 1 2 3 4 5 6 7 8 9 10

6. The child can wear a normal shoe (average 9.6)

Ι Ι Ι Ι Ι Ι Ι Ι Ι Ι Ι

0 1 2 3 4 5 6 7 8 9 10

7. The foot does not supinate in swing phase when walking (average 8.4)

Ι Ι Ι Ι Ι Ι Ι Ι Ι Ι Ι

0 1 2 3 4 5 6 7 8 9 10

8. The heel is in a neutral position (no longer in varus) (average 8.7)

Ι Ι Ι Ι Ι Ι Ι Ι Ι Ι Ι

0 1 2 3 4 5 6 7 8 9 10

9. The foot has 60 degrees of abduction or more (average 7.0)

Ι Ι Ι Ι Ι Ι Ι Ι Ι Ι Ι

0 1 2 3 4 5 6 7 8 9 10

10. The child reports no pain (average 9.3)

Ι Ι Ι Ι Ι Ι Ι Ι Ι Ι Ι

0 1 2 3 4 5 6 7 8 9 10
